# Reduced growth velocity across the third trimester is associated with placental insufficiency in fetuses born at a normal birthweight: a prospective cohort study

**DOI:** 10.1186/s12916-017-0928-z

**Published:** 2017-08-31

**Authors:** Teresa M. MacDonald, Lisa Hui, Stephen Tong, Alice J. Robinson, Kirsten M. Dane, Anna L. Middleton, Susan P. Walker

**Affiliations:** 1 Mercy Perinatal, Mercy Hospital for Women, Melbourne, Australia; 2 Department of Obstetrics and Gynaecology, University of Melbourne, Melbourne, Australia; 3 Translational Obstetrics Group, University of Melbourne, Melbourne, Australia; 4 Department of Obstetrics and Gynaecology, University of Melbourne, Mercy Hospital for Women, 163 Studley Road, Heidelberg, VIC 3084 Australia

**Keywords:** Appropriate-for-gestational-age, Birthweight, Cerebroplacental ratio, Fetal growth restriction, Growth trajectory, Growth velocity, Placental insufficiency, Prenatal, Small-for-gestational-age, Stillbirth, Ultrasonography

## Abstract

**Background:**

While being small-for-gestational-age due to placental insufficiency is a major risk factor for stillbirth, 50% of stillbirths occur in appropriate-for-gestational-age (AGA, > 10th centile) fetuses. AGA fetuses are plausibly also at risk of stillbirth if placental insufficiency is present. Such fetuses may be expected to demonstrate declining growth trajectory across pregnancy, although they do not fall below the 10th centile before birth. We investigated whether reduced growth velocity in AGA fetuses is associated with antenatal, intrapartum and neonatal indicators of placental insufficiency.

**Methods:**

We performed a prospective cohort study of 308 nulliparous women who subsequently gave birth to AGA infants. Ultrasound was utilised at 28 and 36 weeks’ gestation to determine estimated fetal weight (EFW) and abdominal circumference (AC). We correlated relative EFW and AC growth velocities with three clinical indicators of placental insufficiency, namely (1) fetal cerebroplacental ratio (CPR; CPR < 5th centile reflects placental resistance, and blood flow redistribution to the brain – a fetal response to hypoxia); (2) neonatal acidosis after the hypoxic challenge of labour (umbilical artery (UA) pH < 7.15 at birth); and (3) low neonatal body fat percentage (BF%, measured by air displacement plethysmography) reflecting reduced nutritional reserve in utero.

**Results:**

For each one centile reduction in EFW growth velocity between 28 and 36 weeks’ gestation, there was a 2.4% increase in the odds of cerebral redistribution (CPR < 5th centile, odds ratio (OR) (95% confidence interval) = 1.024 (1.005–1.042), *P* = 0.012) and neonatal acidosis (UA pH < 7.15, OR = 1.024 (1.003–1.046), *P* = 0.023), and a 3.3% increase in the odds of low BF% (OR = 1.033 (1.001–1.067), *P* = 0.047). A decline in EFW of > 30 centiles between 28 and 36 weeks (compared to greater relative growth) was associated with cerebral redistribution (CPR < 5th centile relative risk (RR) = 2.80 (1.25–6.25), *P* = 0.026), and a decline of > 35 centiles was associated with neonatal acidosis (UA pH < 7.15 RR = 3.51 (1.40–8.77), *P* = 0.030). Similar associations were identified between low AC growth velocity and clinical indicators of placental insufficiency.

**Conclusions:**

Reduced growth velocity between 28 and 36 weeks’ gestation among fetuses born AGA is associated with antenatal, intrapartum and neonatal indicators of placental insufficiency. These fetuses potentially represent an important unrecognised cohort at increased risk of stillbirth and may warrant more intensive antenatal surveillance.

## Background

One of the most important risk factors for stillbirth is fetal growth restriction (FGR) [1]. In many cases, FGR reflects placental insufficiency, where the placenta is functioning sub-optimally in its role to supply oxygen and nutrients [2], the fetus fails to maintain adequate growth in utero, and is unable to reach its biological growth potential.

Small-for-gestational-age (SGA,  < 10th centile) fetuses, commonly used as a surrogate for FGR, have a three- to four-fold increased risk of stillbirth at every gestation [1, 3, 4]. Being SGA is associated with important antenatal, intrapartum and postpartum indicators of placental insufficiency. Decreased oxygen availability results in the fetus redistributing blood flow to the brain, and placental insufficiency is associated with increased umbilical artery (UA) resistance; these markers can be detected with ultrasound as the ratio of blood flow in the fetal middle cerebral artery (MCA) to that in the UA. Expressed as the cerebroplacental ratio (CPR), this is more sensitive in predicting adverse outcome than either parameter alone [5], and has been proposed as a measure to better identify a fetus failing to achieve their growth potential due to placental insufficiency, irrespective of fetal size [6]. Additionally, placental insufficiency in SGA fetuses may lead to decreased fetal energy reserves. When challenged with the hypoxic stress of labour (uterine contractions limit maternal blood flow to the placenta), there is an increased likelihood of intrapartum acidosis, measured at the time of birth [7, 8]. Finally, decreased fetal energy reserves mean reduced substrate to allow the fetus to store fat, resulting in a lower neonatal fat mass; indeed, there is a strong correlation between being SGA and low body fat percentage (BF%) [9].

Given the increased risk of stillbirth and neonatal morbidity, fetuses suspected to be SGA are intensely monitored antenatally, and are often managed with planned delivery at term. In contrast, fetuses thought to be appropriate-for-gestational-age (AGA, ≥10th centile) are not closely monitored. However, 50% of stillbirths occur in fetuses who are not small, but are in fact AGA [1]. There may be a number of AGA fetuses that slow in their growth trajectory across late pregnancy but who, unlike SGA fetuses, do not fall below the 10th centile threshold by the time of birth. It is possible that such AGA fetuses, demonstrating a low growth velocity, may also be experiencing the effects, and risks, of placental insufficiency, including stillbirth. If so, we might expect them to exhibit the same antenatal, intrapartum and neonatal features of placental insufficiency seen amongst the SGA.

We therefore investigated whether slowing of fetal growth trajectory is associated with indicators of placental insufficiency among AGA infants. Additionally, we determined which clinical thresholds of growth velocity are associated with a significantly increased risk of these measures.

## Methods

### Study design overview

The Fetal Longitudinal Assessment of Growth study was a prospective longitudinal study conducted at the Mercy Hospital for Women, a tertiary maternity hospital in Melbourne, with approximately 6000 births annually.

Fetal size was estimated by ultrasound at 28 and 36 weeks using two parameters, namely the estimated fetal weight (EFW) and the abdominal circumference (AC). For each of these, the gestation-dependent centile was determined. Univariate associations between relative EFW and AC, centile change between 28 and 36 weeks, and clinical indicators of placental insufficiency were then assessed.

The Fetal Longitudinal Assessment of Growth study was designed to investigate whether AGA fetuses that slow in growth trajectory show evidence of placental insufficiency. Therefore, SGA infants (customised birthweight < 10th centile) were excluded from the analysis.

This study was approved by the Mercy Health Research Ethics Committee, Ethics Approval Number R14/12, and written informed consent was obtained from all participants.

### Recruitment

Women were screened for eligibility and invited to participate at their oral glucose tolerance test, universally offered around 28 weeks’ gestation to test for the development of gestational diabetes mellitus. English-speaking women were eligible if they were nulliparous, over 18 years, with a singleton pregnancy and normal mid-trimester fetal morphology examination. Exclusion criteria were known fetal infection, low lying placenta, hypertension, antepartum haemorrhage or ruptured membranes, or EFW < 10th centile at first study ultrasound.

### Ultrasound assessment of fetal size

Ultrasound examinations were performed by one of two experienced operators. The first was performed between 27^+0^ and 29^+0^ weeks’ and the second between 35^+0^ and 37^+0^ weeks’ gestation. For all ultrasounds, a General Electric Voluson 730 (GE Medical Systems, Zipf, Austria) device with 2–7 MHz linear curved-array transducer was used.

Biparietal diameter, head circumference, AC and femur length were recorded using standard biometric planes. Values were measured in triplicate and the mean analysed. EFW was derived from the Hadlock equation utilising all four parameters [10].

Following delivery, ultrasound EFWs and birthweight were customised using the GROW software [11] (http://www.gestation.net/). The GROW software generates a ‘term optimal weight’ based on an optimised fetal weight standard. We used it to adjust for non-pathological factors affecting birthweight, namely maternal height, weight and parity, fetal/infant gender, and exact gestational age. The multiple regression model has a constant to which weight is added or subtracted for each of the variables for which we adjusted. Coefficients for the Australian application of GROW were informed by a local dataset. The mean AC at each ultrasound was converted to a z-score, then centile, using the Chitty AC equation [12].

Treating clinicians were blinded to ultrasound results and were only notified if EFW was below the 10th centile, amniotic fluid index was below the 5th or above the 95th centile [13], UA pulsatility index (PI) was above the 95th centile [14] or MCA PI was below the 5th centile [15], in which case management was at the discretion of the treating team.

### Evaluation of inter- and intra-observer variability

To test for inter-observer variability we performed sub-studies where the two operators who performed all study ultrasounds consecutively scanned the same participant at the same appointment (24 women at 28 weeks’ and 29 women at 36 weeks’ gestation), blinded to the other’s results. Correlation coefficients were > 0.80 for AC and EFW at both gestations, and coefficients of variation were between 3.4% and 6.3% for all individual biometric parameters. Correlation coefficients for intra-observer reliability were ≥ 0.88 for biparietal diameter, head circumference and AC at both gestations. Measurement of femur length had correlation coefficients of 0.82 and ≥ 0.77 at 28 and 36 weeks, respectively. Coefficients of variation for intra-observer reliability analyses were between 3.9% and 5.5% for all parameters at both gestations.

### Calculating fetal growth velocity

To determine the EFW growth velocity, we calculated the change in EFW centile between 28 and 36 weeks by subtracting the 28 week customised EFW centile from the 36 week customised EFW centile. The same process was undertaken to calculate AC growth velocity. Therefore, a fetus whose EFW or AC centile reduced over time had a negative number to describe the EFW or the AC growth velocity respectively, a fetus with no change in centile between ultrasounds had a growth velocity of zero, and a fetus with an increase in centile between ultrasounds had positive growth velocity values. To ensure that the comparison of growth velocity was standardised for the cohort, the change in EFW centile, and in AC centile, between the two ultrasounds were each divided by the exact number of days between examinations, and then multiplied by 56. This facilitated comparison of a standard measure, namely individualised centile change over exactly 8 weeks – defined as the EFW, or AC, third trimester growth velocity.

We analysed the relative fetal growth velocity data for both EFW and AC as separate variables, between 28 and 36 weeks, by (1) analysis of EFW and AC growth velocities as continuous variables and (2) analysis of dichotomous clinically-relevant thresholds of EFW and AC growth velocity. To examine growth velocity as a dichotomous outcome, we primarily defined low growth velocity as an EFW third trimester growth velocity of lower than –30 centiles since ultrasound error in estimating fetal weight is up to 15% [16]. We therefore used a change in customised centile of sufficient magnitude to allow for maximal level of error in either direction at both scans.

### Ultrasound Doppler evaluation

Transabdominal colour Doppler was used to record the UA and MCA waveforms and to calculate the PI for each according to standard protocols [14, 17]. Measurements were taken at times of fetal apnoea and inactivity, with the angle of insonation as close as possible to zero. PI values were measured in triplicate and the mean used. CPR was calculated as mean MCA PI divided by mean UA PI. CPR and MCA PI values were classified as < 5th centile, or not, according to gestation-specific charts [15], and were then converted into gestation-specific multiples of the median (MoM) to facilitate linear analysis. Gestation-specific UA PI values in our cohort were normally distributed and thus were converted to dataset-specific centiles for linear analysis.

### Birthing outcome data

Delivery outcomes and neonatal data were reviewed by a single clinician, blinded to ultrasound growth velocity results. At the time of birth, clinicians were asked to collect paired umbilical cord arterial and venous gas samples from all study participants who underwent labour, where possible. Arterial pH was chosen a priori as our intrapartum indicator of placental insufficiency as it is an objective measure of intrapartum fetal status. A pH cut-off of < 7.15 was chosen as it represents the 5th centile [18], and is a clinically relevant threshold, since pH cut-offs of both 7.10 and 7.20 are associated with increased neurological morbidity risk [19].

### Neonatal body composition assessment

Ponderal index (birthweight (g) × 100)/length^3^ (cm)) was calculated for all study infants. Study newborns also underwent examination of neonatal body composition within 4 days of birth. Neonatal BF% was estimated using triceps and subscapular skinfold thickness and sex-specific equations [20]. Skinfolds were measured in duplicate (triplicate if a difference > 0.4 mm was observed) using a Harpenden skinfold calliper, and the mean value was used. Where possible, BF% was further assessed by air displacement plethysmography (ADP), the most robust and reproducible assessment of neonatal BF% [21], using a PEA POD (COSMED, Concord, CA, USA) device according to the manufacturer’s instructions.

### Sample size calculation

Our previous pilot study found that 19% of fetuses exhibited low EFW third trimester growth velocity (lower than –30 centiles) and were born AGA [22]. This pilot study observed a four-fold increase in intrapartum compromise among AGA fetuses with low growth velocity. For sample size calculation, we assumed a 6% background rate of neonatal acidosis (UA pH < 7.15) amongst the AGA [8] and aimed to detect a 3.8-fold increased rate of this outcome amongst those with low EFW third trimester growth velocity. With 80% power and α = 0.05, this would require 65 cases of low EFW third trimester growth velocity among AGA fetuses. We therefore required a total of 342 participants to complete both ultrasounds, estimating that, of these, 65 (19%) would be AGA with low EFW growth velocity, 34 (10%) would be SGA infants at birth and excluded from the study, and the remaining 243 (71%) would be AGA fetuses who maintained their in utero growth velocity.

### Statistical analysis

Maternal characteristics and birth outcome data were compared between recruited participants and eligible women not recruited as well as between cases of low EFW third trimester growth velocity and the remainder of the cohort using non-paired t-test (normally distributed) or Mann–Whitney test (not normally distributed) for continuous data, and χ^2^ or Fisher’s exact tests for categorical analyses. The D’Agostino and Pearson omnibus normality test was utilised to determine the distribution of continuous data [23].

We assessed the relationships between EFW and AC growth velocities, and indicators of placental insufficiency, in three ways: (1) we plotted the biological relationships between EFW and AC growth velocities as continuous variables against our continuous outcomes using linear regression; (2) we analysed the relationships between growth velocities as continuous variables and our pre-defined dichotomous indicators of placental insufficiency using logistic regression – this determined the odds of each given outcome per centile decrease in third trimester growth velocity; and (3) we assessed dichotomous clinically relevant thresholds, starting with EFW and AC growth velocities of < –30 centiles compared to the remainder of the cohort, to ascertain the relative risks of our pre-defined indicators of placental insufficiency using Fisher’s exact test.

### Analysis of growth velocity and antenatal evidence of placental insufficiency

The relationship between fetal growth velocity and antenatal evidence of placental insufficiency was examined using (1) linear regression to assess correlations between EFW, and AC, third trimester growth velocities, and 36-week CPR MoM (as well as the individual parameters MCA PI MoM and UA PI centile); (2) univariate logistic regression to assess the relationships between EFW, and AC, third trimester growth velocities each as individual variables, and low CPR and low MCA PI (<5th centile [15]) as individual variables (there were no UA PI values > 95th centile [14] in our cohort, so logistic regression was not performed for this parameter); and (3) Fisher’s exact test to interrogate clinical thresholds (starting with < –30 centiles) to dichotomise the cohort, and elucidate the associated risk of low CPR.

### Analysis of growth velocity and intrapartum evidence of placental insufficiency

The relationship between growth velocity and intrapartum evidence of placental insufficiency was examined using (1) logistic regression to assess the relationships between EFW, and AC, third trimester growth velocities, each as individual variables, and UA pH < 7.15 amongst those who underwent labour and (2) Fisher’s exact test to interrogate the risks of intrapartum acidosis associated with dichotomous clinical thresholds of growth velocity.

### Analysis of growth velocity and neonatal evidence of placental insufficiency

The relationship between growth velocity and neonatal evidence of placental insufficiency was examined using (1) linear regression to assess the correlations between EFW, and AC, third trimester growth velocities and all body composition parameters (ponderal index, skinfold BF% and ADP BF%); (2) univariate logistic regression analysis to assess the relationships between EFW and AC, third trimester growth velocities, and low ADP BF% (defined as < 4.2% for males and < 5.8% for females, previously found to equate to more than one standard deviation (SD) below the mean [24]); and (3) Fisher’s exact test to interrogate dichotomous clinical thresholds of growth velocity and their associated risks of low ADP BF%.

Statistical analysis was performed using GraphPad Prism version 6.00 for Windows (GraphPad Software, La Jolla, CA, USA, http://www.graphpad.com/), except for logistic regression, which was performed using R, version 3.3.2 (64-bit).

## Results

### Study participants

**Fig. 1:**
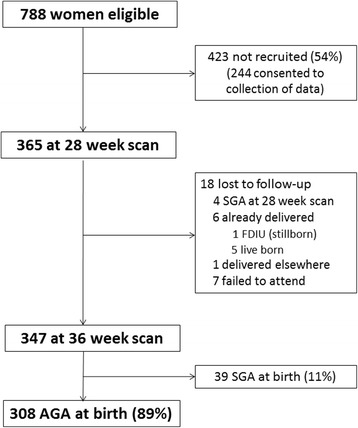
Study profile

Between February 2015 and February 2016, 365 (46.3%) of 788 eligible women were recruited. Of the 365 participants enrolled, 347 completed both study ultrasounds allowing calculation of third trimester fetal growth velocities. Of these, 39 (11.2%) infants were SGA according to customised birthweight centile and excluded, such that 308 AGA infants (88.8%) were included in the analysis (Fig. 1).

The characteristics of the 308 participants are shown in Table 1. When participants exhibiting low third trimester growth velocity (< –30 EFW centiles) were compared to the remainder of the cohort, there were no significant differences in maternal characteristics or onset or mode of delivery, except for booking body mass index. The low EFW third trimester growth velocity cohort had a median body mass index of 24.8 kg/m^2^, compared to 27.0 kg/m^2^. Low third trimester growth velocity infants were significantly smaller than those of the remainder of the cohort (mean birthweight 3160 g vs. 3453 g), and had correspondingly lower median customised birthweight centile (35.0 vs. 52.9).

**Table 1:** Maternal characteristics and delivery outcomes – maternal characteristics and delivery outcomes of participants overall and comparison between low third trimester growth velocity and the remainder of the cohort

	Total analysis cohort (n = 308)	Low third trimester growth velocity^a^(n = 26)	Normal growth velocity (n = 282)	*P*
Age, years	31.0 (4.2)	31.9 (2.5)	30.9 (4.3)	0.23
Booking BMI, kg/m^2^	26.9 (24.4–30.1)	24.8 (23.1–28.5)	27.0 (24.6–30.1)	0.02
Smoking status				
Current smoker Ex-smoker Never Unknown	5 (1.6%) 84 (27.3%) 218 (70.8%) 1 (0.3%)	0 (0.0%) 4 (15.4%) 22 (84.6%) 0 (0.0%)	5 (1.8%) 80 (28.4%) 196 (69.5%) 1 (0.4%)	0.26
Gestational hypertension or preeclampsia	42 (13.6%)	5 (19.2%)	37 (13.1%)	0.37
GDM	38 (12.3%)	2 (7.7%)	36 (12.8%)	0.75
Onset of delivery				
Induction of labour Spontaneous labour No labour	147 (47.7%) 139 (45.1%) 22 (7.1%)	9 (34.6%) 16 (61.5%) 1 (3.8%)	138 (48.9%) 123 (43.6%) 21 (7.4%)	0.21
Mode of delivery				
Normal vaginal delivery Instrumental delivery Emergency caesarean Elective caesarean	117 (38.0%) 101 (32.8%) 70 (22.7%) 20 (6.5%)	14 (53.8%) 7 (26.9%) 4 (15.4%) 1 (3.8%)	103 (36.5%) 94 (33.3%) 66 (23.4%) 19 (6.7%)	0.37
Birthweight, g	3453 (431.0)	3160 (396.8)	3480 (424.6)	0.0003
Customised birthweight centile	49.9 (26.7–72.6)	35.0 (22.7–42.8)	52.9 (27.6–74.2)	0.007
Gestational age at birth, weeks	39.9 (38.9–40.6)	39.7 (37.7–40.3)	40.0 (39.0–40.7)	0.03

Data presented as mean (standard deviation) or median (interquartile range) depending on distribution for continuous variables, and as number (%) for categorical variables

*BMI* body mass index, *GDM* gestational diabetes mellitus

^a^Low third trimester growth velocity is an EFW growth velocity of < –30 centiles over 8 weeks

Overall, 423 eligible women were not recruited. Of these, 179 were not located at the time of their glucose tolerance test or declined participation. The remaining 244 did not participate but agreed to the collection of outcome data. To evaluate the possibility of recruitment bias, we compared the maternal characteristics and delivery outcomes of these 244 non-participants and the 365 recruited women and found no significant differences (Table 2). Induction of labour and mode of delivery rates for both groups were consistent with those of all nulliparous women at our institution during the corresponding period. Thus, there was no evidence of recruitment or selection bias.

**Table 2:** Assessment for recruitment bias – demographic and delivery characteristics of recruited participants compared to eligible women who were not recruited

	Recruited (n = 365)	Not recruited (n = 244)	*P*
Age, years	30.9 (4.1)	31.6 (4.2)	0.05
Booking BMI, kg/m^2^	23.7 (21.5–26.9)	23.5 (21.5–26.2)	0.26
Smoking status			
Current smoker Ex-smoker Never	7 (1.9%) 95 (26.1%) 262 (72.0%)	7 (2.9%) 51 (20.9%) 186 (76.2%)	0.28
Gestational hypertension or pre-eclampsia	53 (14.6%)	42 (17.3%)	0.37
GDM	47 (12.9%)	26 (10.7%)	0.45
Onset of delivery			
Induction of labour Spontaneous labour No labour	177 (48.6%) 155 (42.6%) 32 (8.8%)	104 (42.8%) 117 (48.1%) 22 (9.1%)	0.35
Mode of delivery			
Normal vaginal delivery Instrumental delivery Emergency caesarean Elective caesarean	137 (37.6%) 118 (32.4%) 80 (22.0%) 29 (8.0%)	90 (37.0%) 76 (31.3%) 58 (23.9%) 19 (7.8%)	0.96
Birthweight, g	3331 (514.5)	3308 (526.0)	0.87
Birthweight centile	39.0 (18.1–69.6)	40.6 (20.8–64.9)	0.83
SGA, < 10th centile	45 (12.4%)^a^	28 (11.5%)	0.80
Severe SGA, < 3rd centile	13 (3.6%)^a^	9 (3.7%)	1.00
Gestational age at delivery, weeks	39.7 (38.7–40.6)	39.7 (38.7–40.6)	0.80

Data presented as mean (standard deviation) or median (interquartile range) depending on distribution for continuous variables, and as number (%) for categorical variables

*BMI* body mass index, *GDM* gestational diabetes mellitus, *SGA* small-for-gestational-age

^a^The 45 cases where SGA (customised birthweight < 10th centile) infants were delivered were excluded from the final analysis

### Antenatal Doppler parameters according to third trimester fetal growth velocity

**Fig. 2:**
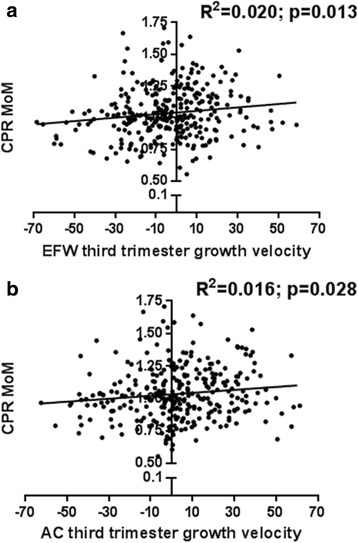
Cerebroplacental ratio (CPR) multiples of the median (MoM) according to third trimester growth velocity. **a** CPR MoM according to estimated fetal weight third trimester growth velocity; (**b**) CPR MoM according to abdominal circumference third trimester growth velocity

We first examined whether a decrease in EFW or AC centiles between 28 and 36 weeks’ gestation is associated with a decrease in the CPR at 36 weeks. A lower CPR can occur when there is increased redistribution of fetal circulation to the brain as an adaptive response to placental insufficiency and/or increased resistance in the UA due to placental dysfunction. EFW and AC third trimester growth velocities were both significantly correlated with 36-week CPR MoM (Fig. 2a, b). This suggests a direct relationship between decreasing EFW and AC, growth velocities and a lower CPR, where the lower the growth velocity, the greater the degrees of cerebral redistribution and placental resistance. Linear regression analysis of fetal growth velocities and the individual parameters of the CPR – the MCA PI MoM and the UA PI centiles – demonstrated decreasing MCA PI MoM and increasing UA PI centiles with decreasing growth velocities, but these correlations were not significant (data not shown).

A lower EFW growth velocity was also significantly associated with low 36-week CPR (<5th centile [15]). For each single centile decrease in EFW third trimester growth velocity, the odds of low CPR at 36 weeks’ gestation increased by 2.4% (Table 3). There were non-significant trends seen towards increased odds of CPR < 5th centile for each centile decrease in AC third trimester growth velocity and increased odds of MCA PI < 5th centile [15] for each centile decrease in EFW and AC third trimester growth velocities (Table 3).

**Table 3:** Odds of placental insufficiency measures, per centile decrease in EFW/AC third trimester growth velocity

Outcome	Growth parameter	Odds ratio (95% CI) of outcome per centile decrease in third trimester growth velocity	*P*
CPR < 5th centile (n = 305)	EFW	1.024 (1.005–1.042)	0.01
	AC	1.015 (0.997–1.032)	0.10
MCA PI < 5th centile (n = 308)	EFW	1.005 (0.987–1.025)	0.57
	AC	1.013 (0.995–1.031)	0.17
UA pH < 7.15 at birth (n = 241)	EFW	1.024 (1.003–1.046)	0.02
	AC	1.022 (1.002–1.041)	0.03
ADP low body fat percentage (n = 137)	EFW	1.033 (1.001–1.067)	0.047
	AC	1.036 (1.005–1.068)	0.02

*AC* abdominal circumference, *ADP* air displacement plethysmography, *CI* confidence interval, *CPR* cerebroplacental ratio, *EFW* estimated fetal weight, *MCA PI* middle cerebral artery pulsatility index, *UA* umbilical artery

We next examined the rates of low CPR (<5th centile [15]) when the cohort was dichotomised as to whether the fetus had low EFW or AC growth velocity of < –30 centiles between 28 and 36 weeks or not. A low CPR was significantly more common, with a relative risk (RR) of 2.8, in fetuses with low third trimester EFW growth velocity of < –30 centiles. A low AC third trimester growth velocity of < –30 centiles trended towards an increased incidence of low CPR, but did not reach statistical significance (Table 4).

**Table 4:** Relative risk of measures of placental insufficiency when growth velocity cut-off thresholds dichotomise the cohort

Outcome	Definition of low growth velocity	Growth velocity		RR (95% CI) if low velocity	*P*
		Low n (%)	Not low n (%)		
CPR < 5th centile (n = 305)	EFW < –30 centiles	6/26 (23.1)	23/279 (8.2)	2.80 (1.25–6.25)	0.03
	AC < –30 centiles	4/25 (16.0)	25/280 (8.9)	1.79 (0.68–4.74)	0.28
UA pH < 7.15 at birth (n = 241)	EFW < –30 centiles	4/19 (21.1)	20/222 (9.0)	2.34 (0.89–6.14)	0.11
	AC < –30 centiles	4/21 (19.0)	20/220 (9.1)	2.10 (0.79–5.56)	0.14
	EFW < –35 centiles	4/13 (30.8)	20/228 (8.8)	3.51 (1.40–8.77)	0.03
	AC < –35 centiles	4/15 (26.7)	20/226 (8.8)	3.01 (1.18–7.70)	0.049
ADP low body fat percentage (n = 137)	EFW < –30 centiles	1/11 (9.1)	11/126 (8.7)	1.04 (0.15–7.34)	1.00
	AC < –30 centiles	4/8 (50.0)	8/129 (6.2)	8.06 (3.07–21.16)	0.002

*AC* abdominal circumference, *ADP* air displacement plethysmography, *CI* confidence interval, *CPR* cerebroplacental ratio, *EFW* estimated fetal weight, *RR* relative risk, *UA* umbilical artery

Therefore, we conclude that decreasing fetal EFW centiles, and to a lesser extent decreasing AC centiles, between 28 and 36 weeks’ gestation are significantly associated with a combination of increased redistribution of blood flow to the fetal brain and increased placental resistance at 36 weeks, both of which are antenatal indicators of placental insufficiency.

### Intrapartum outcomes according to third trimester fetal growth velocity

A fetus suffering placental insufficiency has lower energy and oxygen reserves. Thus, when confronted with the hypoxic challenge of labour, they are more likely to develop intrapartum acidosis. We analysed the relationship between EFW and AC growth velocities and low cord UA pH (<7.15) at birth. Overall, 288 participants underwent labour and, of these, 241 (83.7%) had a cord UA pH recorded at birth available for this analysis.

When EFW and AC third trimester growth velocities were analysed as continuous variables, lower EFW and AC growth velocities were both significantly associated with UA pH < 7.15 at birth. For each centile decrease in EFW third trimester growth velocity, the odds of UA pH < 7.15 increased by 2.4%. For each centile decrease in AC third trimester growth velocity, the odds of UA pH < 7.15 increased by 2.2% (Table 3). This suggests that the lower the growth velocity, the greater the degree of reduced placental reserve, increasing the odds of developing acidosis under the hypoxic challenge of labour.

We next examined the rates of intrapartum acidosis (UA pH < 7.15) when the cohort was dichotomised according to EFW or AC third trimester growth velocity of < –30 centiles or not. The rate of UA pH < 7.15 was increased in fetuses with an EFW growth velocity of < –30 centiles compared to the rest of the cohort (21% vs. 9%), but this just failed to achieve statistical significance. Third trimester EFW growth velocity of < –35 centiles, however, was associated with a 3.5-fold increase in the risk of intrapartum acidosis (RR (95% confidence interval) = 3.5 (1.4–8.8), *P* = 0.03). Acidosis in labour was also significantly three times more common in those with a low AC third trimester velocity of < –35 centiles (Table 4).

### Neonatal body composition according to third trimester fetal growth velocity

Ponderal index was calculated for all study infants, whereas neonatal skinfold measurements to estimate BF% were performed on 271 (88.0%) infants and 137 (44.5%) were assessed by ADP. We examined whether a decrease in EFW or AC centiles between 28 and 36 weeks’ gestation is associated with lower neonatal body fat. In the presence of placental insufficiency, a fetus has a decreased nutrient and oxygen supply and will therefore have less substrate available for fat deposition. Thus, low body fat percentage is a neonatal indicator of placental insufficiency.

**Fig. 3:**
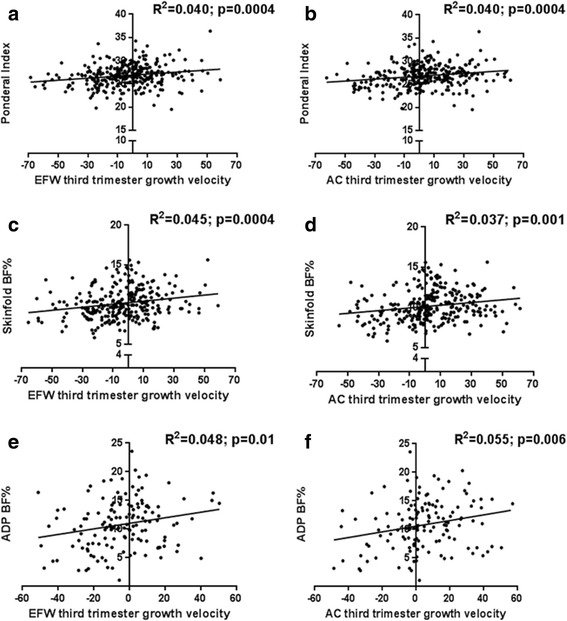
Neonatal ponderal index and body fat percentage (BF%) according to third trimester growth velocity. **a** Ponderal index according to estimated fetal weight (EFW) third trimester growth velocity. **b** Ponderal index according to abdominal circumference (AC) third trimester growth velocity. **c** Skinfold BF% according to EFW third trimester growth velocity. **d** Skinfold BF% according to AC third trimester growth velocity. **e** Air displacement plethysmography (ADP) BF% according to EFW third trimester growth velocity. **f** ADP BF% according to AC third trimester growth velocity

When linear analysis was performed, EFW and AC third trimester growth velocities were both significantly correlated with all measures of neonatal fat deposition, namely ponderal index, skinfold BF% and ADP BF% (Fig. 3a–f). This suggests a direct relationship between decreasing EFW and AC growth velocities and lower fetal energy reserves and substrate supply with which to store fat in utero.

Where available, we used the most robust method of neonatal fat assessment (PEA POD ADP device) to determine the neonatal BF%. We defined low ADP BF% as percentages previously found to represent more than one SD below the mean (<4.2% for males, < 5.8% for females) as these cut-offs have the greatest sensitivity for neonatal morbidity [24]. Lower EFW and AC third trimester growth velocities were both significantly associated with low ADP BF%. For each centile decrease in EFW third trimester growth velocity, the odds of low ADP BF% increased by 3.3%, and for each centile decrease in AC third trimester growth velocity, the odds of low BF% increased by 3.6% (Table 3).

We examined the rates of low ADP BF% when the cohort was dichotomised according to EFW or AC third trimester growth velocity, starting with a threshold of < –30 centiles. When EFW growth velocity was tested, a clinical threshold could not be elucidated that significantly increased the RR of low ADP BF%. However, a low ADP BF% was significantly more common in neonates with a low third trimester AC growth velocity of < –30 centiles, with a RR of 8.1 (Table 4).

Thus, we conclude that low EFW and AC fetal growth velocity between 28 and 36 weeks’ gestation is associated with a lower ponderal index and lower neonatal BF%, suggesting neonatal evidence of placental insufficiency.

## Discussion

### Main findings

In this prospective longitudinal study, we have found that AGA fetuses who display low third trimester growth velocity exhibit antenatal, intrapartum and neonatal features suggestive of placental insufficiency. We report significant correlations between growth velocity and low CPR, reflective of fetal cerebral blood flow redistribution and increased placental resistance during pregnancy, development of acidosis under the hypoxic challenge of labour and reduced neonatal body fat stores. These clinical indicators of placental insufficiency are also associated with fetuses born SGA [5, 8, 9, 25], who have three- to four-fold increased risk of stillbirth [1, 3, 4]. Thus, the implications of our findings are that AGA fetuses that decline in growth trajectory may also be suffering from placental insufficiency, placing them at increased risk of stillbirth.

### Interpretation of the findings and comparison with other studies

The only other study to examine fetal growth velocity exclusively among AGA fetuses was our previous pilot study [22]. In 48 participants, our pilot study analysed the relationships between fetal growth velocity and both cerebral redistribution and operative delivery for suspected intrapartum compromise, but did not assess the associations with UA pH < 7.15 after labour, or neonatal BF% [22]. Our pilot study led us to undertake this larger study, with expanded power and examination of more sophisticated measures of placental insufficiency.

Other studies have reported associations between reduced growth velocity and adverse perinatal outcome [26–29], but have included SGA infants, who are known to have an increased risk of stillbirth. Notably, the Pregnancy Outcome Prediction (POP) study found low AC growth velocity to predict for adverse outcome in SGA fetuses, but not in the AGA [26]. However, in contrast to our study, the POP study did not investigate for cerebral redistribution, did not assess neonatal body composition, and did not evaluate EFW growth velocity. Furthermore, the POP study did not examine AC growth velocity as a continuous variable, while we consistently found significantly increased odds of placental insufficiency indicators when AC, and EFW, growth velocities were analysed in this way. A recent, large retrospective study also investigated AC growth velocity and the CPR. In keeping with our results, a low CPR was significantly associated with a low AC growth velocity, birthweight centile and operative delivery for presumed fetal compromise, even when analysis was confined to AGA fetuses [30].

That AGA fetuses with a declining growth trajectory may be at increased risk of stillbirth is supported by epidemiological data. The lowest rate of perinatal death occurs amongst those with birthweight between the 75th and 90th centiles [31, 32], with perinatal mortality significantly increasing at every centile below the 50th. Compared to the 75th to 90th centile group, the adjusted odds for perinatal death are doubled for those with birthweight of 10th to 25th centile, and for those born between the 25th and 50th centiles the adjusted odds ratio is increased, at 1.58 [31]. This stepwise increase in perinatal death with falling birthweight centile, even among the AGA, may in part be explained by placental insufficiency causing reduced growth velocity. That placental insufficiency plays a role is further supported by a progressive decrease in CPR seen with falling birthweight centile [33], mirroring perinatal death rates [6]. In the setting of poor placental function, fetal growth slows, but whether the final birthweight falls below the 10th centile depends upon the starting fetal weight centile, the severity of placental insufficiency and the duration of fetal exposure to placental insufficiency (determined by gestation at onset and birth).

It is not surprising that 81% (21/26) of the fetuses exhibiting low EFW third trimester growth velocity in this study had a customised birthweight between the 10th and 50th centiles – placing them within the range of significantly increased risk of perinatal death. However, 40% of all fetuses would be expected to be born with a birthweight within this range, so this categorisation would perform poorly as a predictive test of adverse outcome. In contrast, an EFW growth velocity of < –30 centiles over 8 weeks occurred in only 8.4% of AGA fetuses. Therefore, this may be a clinical tool with better predictive value in detecting the AGA fetus suffering placental insufficiency, at risk of stillbirth.

The clinical significance of reduced fetal growth velocity has been addressed in a recently published expert consensus definition of FGR. The International Society for Ultrasound in Obstetrics and Gynaecology definition of FGR now includes “AC/EFW crossing centiles” (defined as > 2 quartiles or 50 centiles) as long as it occurs in a SGA fetus (<10th centile EFW or AC), and/or in conjunction with a low CPR or elevated UA PI [34]. However, our findings support a more conservative threshold – of a fetus declining by an equivalent of > 30 EFW or AC centiles over 8 weeks – given the significant associations with robust measures of placental insufficiency we have demonstrated. A > 50 centile reduction occurred in only eight (2.6%) of our AGA study participants, and if the consensus definition’s SGA and/or abnormal Doppler criteria were also applied to our initial cohort, then only 1.4% (5/347) of fetuses would have been detected. This may mean that a number of AGA fetuses with placental insufficiency, who may be at risk of stillbirth, would be missed if a > 50 centiles threshold was utilised.

### Strengths and limitations

A major strength of this study is the examination of multiple and diverse measures of placental insufficiency across the antenatal, intrapartum and neonatal periods. The antenatal feature of placental insufficiency used was the CPR at 36 weeks. The fetus adapts to hypoxia by preferentially perfusing the cerebral vasculature, which, together with what may be subtle increases in placental resistance, result in a low CPR. The CPR is the most sensitive ultrasound measure in late pregnancy [5] and is associated with the development of fetal decompensation and acidosis in labour [30, 35–39], neonatal unit admission [36], and stillbirth [40]. Umbilical artery pH is the most reliable and objective measure of intrapartum fetal compromise, and has the best correlation with key perinatal outcomes [41], including neonatal mortality, hypoxic ischaemic encephalopathy and cerebral palsy [19]. Finally, placental insufficiency results in reduced substrate supply and neonatal body fat among SGA infants [9]. Ponderal index and skinfold thickness are widely used as anthropometric methods to diagnose impaired fetal growth, but ADP used to assess BF% is the gold standard for the assessment of newborn body composition [21]. ADP is more accurate than dual-energy X-ray absorptiometry [42, 43], and demonstrates better prediction of neonatal morbidity than birthweight centile [24].

There were a number of other strengths to our study. This was a prospective study of large numbers; customisation of EFW and birthweight centiles was performed as customised centiles share a stronger association with adverse perinatal outcomes than population references [44]; we interrogated fetal growth velocity using two approaches (EFW and AC); we obtained evidence to counter the possibility of selection or recruitment bias; and we demonstrated low inter- and intra-observer variation between ultrasound operators.

The main limitation of our study is that it was not powered to detect important but uncommon perinatal outcomes such as stillbirth or significant neonatal morbidity. In addition, the rate of low third trimester growth velocity (EFW velocity of < –30 centiles) was 7.6% among our entire cohort of AGA and SGA fetuses, which was lower than the 19% demonstrated in our pilot study [22]. Further, we did not manage to collect an umbilical arterial cord gas from every study participant. Finally, the rate of neonatal acidosis was higher than anticipated (9% vs. 6%) among AGA infants who maintained their growth velocity [8]. As such, we were underpowered to detect the 3.8-fold increased rate of neonatal acidosis among the low growth velocity cohort as originally intended. However, a trend towards this outcome was seen, and we were able to demonstrate a significant 3.5-fold increased neonatal acidosis risk among those with a EFW growth velocity of < –35 centiles. Overall, our cohort was small, and thus our results should be validated in a larger study.

### Clinical and research implications

Our data raises the possibility that serial ultrasound growth assessments may have a role in the clinical management of pregnancy. However, there remains debate about universal ultrasound for all pregnant women at 28 and 36 weeks’ gestation given the significant cost implications and that we are yet to demonstrate improved clinical outcomes. However, for women already assessed by serial ultrasound due to risk factors, our data suggests that AGA fetuses who demonstrate a significant decline in growth trajectory may warrant increased surveillance and management, as might be instituted in cases of a SGA fetus with presumed placental insufficiency. Furthermore, our data may be used to inform the design of an appropriately powered interventional study to elucidate the value of enhanced fetal surveillance and timely birth for AGA fetuses with low third trimester growth velocity.

## Conclusion

AGA infants who display low growth velocity in late pregnancy exhibit antenatal, intrapartum and neonatal features suggestive of in utero placental insufficiency typically associated with SGA fetuses. This study has identified a cohort of AGA fetuses that may plausibly be at risk of stillbirth. Further research is required to determine whether this group may benefit from increased surveillance and timely delivery.

## Abbreviations

AC: Abdominal circumference
ADP: Air displacement plethysmography
AGA: Appropriate-for-gestational-age
BF%: Body fat percentage
CPR: Cerebroplacental ratio
EFW: Estimated fetal weight
FGR: Fetal growth restriction
MCA: Middle cerebral artery
MoM: Multiples of the median
PI: Pulsatility index
POP: Pregnancy outcome prediction
RR: Relative risk
SD: Standard deviation
SGA: Small-for-gestational-age
UA: Umbilical artery
